# Anomaly Detection of Water Level Using Deep Autoencoder

**DOI:** 10.3390/s21196679

**Published:** 2021-10-08

**Authors:** Isack Thomas Nicholaus, Jun Ryeol Park, Kyuil Jung, Jun Seoung Lee, Dae-Ki Kang

**Affiliations:** 1 Department of Computer Engineering, Dongseo University, Busan 47011, Korea; isaactnicholaus@gmail.com; 2 Buzzni AI Lab, Seoul 08788, Korea; parker@buzzni.com; 3 JCMEEK, Seoul 07591, Korea; jcmeek147@jcmeek.com; 4 Infranics R&D Center, Seoul 07994, Korea; jun.lee@infranicsmail.com

**Keywords:** anomaly detection, deep autoencoder, time-series

## Abstract

Anomaly detection is one of the crucial tasks in daily infrastructure operations as it can prevent massive damage to devices or resources, which may then lead to catastrophic outcomes. To address this challenge, we propose an automated solution to detect anomaly pattern(s) of the water levels and report the analysis and time/point(s) of abnormality. This research’s motivation is the level difficulty and time-consuming managing facilities responsible for controlling water levels due to the rare occurrence of abnormal patterns. Consequently, we employed deep autoencoder, one of the types of artificial neural network architectures, to learn different patterns from the given sequences of data points and reconstruct them. Then we use the reconstructed patterns from the deep autoencoder together with a threshold to report which patterns are abnormal from the normal ones. We used a stream of time-series data collected from sensors to train the model and then evaluate it, ready for deployment as the anomaly detection system framework. We run extensive experiments on sensor data from water tanks. Our analysis shows why we conclude vanilla deep autoencoder as the most effective solution in this scenario.

## 1. Introduction

Problems involving anomaly detection such as frauds [1], medical application faults [2], security systems intrusion [3], system faults [4], and similar issues [5,6] are usually challenging because of their data imbalance nature. Normally anomalies rarely happen, which leads to a lack of sufficient anomaly data for eliciting relevant information essential for analysis and generalization of anomalies behaviors. This is important because, in many safety-critical applications, failure to predict and capture anomalies at the right moments is likely to result in catastrophic outcomes [7]. Therefore, efficient automated anomaly detectors that do not require human supervision are needed to mitigate the issues and elevate safety.

Extensive studies [8,9,10,11] analyzing and classifying outliers have been conducted targeted for various real-world applications. Specifically, anomaly detection systems incorporated with machine learning and deep learning algorithms [12,13,14,15,16] have been widely investigated. For instance, Ding et al. [17] use Bayesian network to capture anomalies in real-time multivariate time-series. Li et al. [18] and Oza et al. [19] adopted one-class Support Vector Machine(SVM). Deep-learning-based methods have exhibited superior (or at least comparable) abilities over classical approaches in various fields, such as dimension reduction that take over classical approaches such as PCA [20,21].

Basically, dimension reduction tries to find a subspace that can optimally represent the normal data. Then, the data are projected to this optimal subspace, and data points with high reconstruction errors exceeding the user-supplied threshold can be assumed to be anomalies [22]. Autoencoder exhibits excellent dimensionality reduction, making this method the promising candidate to be the main component of the anomaly detection system [23]. Unlike PCA that uses linear transform [20], autoencoder-based approaches can perform non-linear transformations with their multiple layers and non-linear activation functions, thus our proposed method manifests its merits when the water-level data patterns are naturally complex and non-linear.

Detection of anomalous patterns using autoencoder follows the main idea of dimension reduction-based anomaly detection techniques, which is based on reconstruction error. Therefore, the training of autoencoder targets on decreasing the reconstruction error (e.g., mean squared error or MSE) on the normal data. Then a certain threshold can be applied to the reconstruction error for capturing anomalies. Please note that reconstruction error will become high for the abnormal input while it will be low for the normal input, considering the underlying patterns learned from the normal training data.

In this paper, we present a novel time-series deep autoencoder-based anomaly detection technique for water-level anomalies. First, we designed a time-series data preprocessing pipeline to handle training and testing data preparation. Then, we trained an autoencoder neural network-based model on preprocessed normal data only using two different procedures as discussed in Section 3.7. After that, we evaluated the trained model on normal and abnormal data to produce a practical and reliable discriminator of regular and irregular patterns over great extents of time-series data. From the evaluation, it can be seen that we successfully produced a simple but effective machine learning system that quickly learns normal time-series data complex patterns and exhibits excellent performance on unseen data.

## 2. Related Work

The anomaly detection problem [1,2,3,4,5,6] has been widely explored in various fields, but it is under-explored for water monitoring system. However, this problem is essential to safeguard the facilities responsible for monitoring and controlling water levels. In this section, we summarize the recent research on the anomaly detection for water monitoring system. To the best of our knowledge there are no prior work that adopt deep autoencoder for water-level anomaly detection, and thus we employed this technique to handle both point and collective anomaly. A few previous studies focused on similar problems will be described in the next several paragraphs.

Autoencoder approaches are machine learning techniques that are widely used in anomaly detection. Over the years, there has been a huge number of papers in the machine learning community exploring different areas of application of autoencoder approaches. For instance, Al-amri et al. [24] presented an intensive review of machine learning and deep learning for anomaly detection in IoT data. In the case of autoencoders for anomaly detection, the authors discussed varieties of autoencoders such as the stacked denoising autoencoder [25] and Streaming Autoencoder (SA) [26]. Rosso et al. [27] proposed an autoencoder based on 1D Convolutional Neural Network (CNN) layers and validated their approach on multivariate time-series data. Tien et al. [28] employed autoencoder for dimensionality reduction, learning significant features and went even further to use autoencoder for transfer learning. On the other hand, Shvetsova et al. [29] introduced a classical autoencoder-based approach with their new training pipeline approach design to handle complex and high-resolution images. Shvetsova et al. work targeted abnormalities in images obtained from medical domain. For deeper investigation on autoencoders for anomaly detection, the work done by Finke et al. [30] intensively discussed both capabilities of the autoencoder approaches and their limitations.

Buras and Donado [31] focused on the problem of identifying a wastewater pollutant and localizing its source point in the wastewater network, given a time-series of wastewater measurements collected by sensors positioned across the sewer network. The authors divided the problem into two sub-problems. As the related work to our work, we will only describe the first sub-problem. The first sub-problem deals with detecting and identifying the flowing pollutants in wastewater, i.e., assessing whether a given time-series corresponds to a contamination event and determining what polluting substance caused it. The authors applied random forest [32] classifiers to solve the problem. The authors considered to employ the random forest classifiers because of the capability of the algorithm to merge the predictions from multiple decision trees that leads higher final accuracy of the model. However, the random forest algorithm is a supervised machine learning algorithm, and thus it is inappropriate to employ this technique for our problem of water-level anomaly detection. This is because we need the algorithm to learn from normal data only and later we apply the generated model on normal and abnormal data points. Compared with their approach, our proposed method is an unsupervised deep learning algorithm, which can efficiently learn complex patterns from normal data only and then exhibits excellent performance on the unseen data that contains both normal and abnormal data points.

Tan et al. [33] designed and used hierarchical models to build a dual-stage cascade one-class support vector machine (OCSVM) for water-level monitor systems. The work focused on point and collective anomaly [34] detection. In the first stage, 1-g OCSVM learns directly on a single observation to detect point anomaly. For the second stage, n-gram OCSVM learns from the constructed n-gram feature vectors based on the historical data to discover any collective anomaly where the pattern from the n-gram failed to conform to the expected normal pattern. Their work successfully optimized hyperparameters of different window sizes and statistical measurements for the effective solution to deal with huge amount of data, and demonstrated their technique targeted to point and point collective anomaly. Despite the achievement demonstrated by the OCSVM, the technique is not suitable for large datasets compared with our technique (i.e., deep learning-based technique) which can handle huge data. Additionally, the authors pointed out that the OCSVM is likely to overfit if the window size is excessively large (i.e., the maximum window size was 300) which is not the problem to our deep autoencoder model, since it can manage up to window size of 600.

Kulanuwat et al. [35] proposed an approach to detect outliers in the water-level data. They developed a median-based statistical outlier detection approach using a sliding window technique. In their work, they used simple but effective interpolation techniques such as linear interpolation and spline interpolation to fill anomalies and missing values. For training the n-gram data from the interpolation, they chose advanced techniques including long short-term memory (LSTM). The authors adopted median absolute deviation (MAD) technique, which is insensitive to large deviations of the time-series data. However, high sensitivity to any deviation is crucial for our problem because even a single point deviates from the norm should be captured and reported as anomaly.

Chachula et al. [36] implemented and evaluated a data fusion system that transforms the time-series of sensor measurements into a collection of source-localized discharge events. Based on experiment results, the authors have shown that the proposed framework is an efficient solution for pollution source localization because it can narrow down the number of sources of pollution nodes and therefore achieved faster sensor observations processing (i.e., 100 observations per second). However, their approach does not consider deep learning for disaster detection which limits its capability to work with complex data and complicated utility network structures. Moreover, this approach is proposed for specific water monitoring and systematic sensor data management for the utility network. Unlike their approach, we employed deep learning, which can be flexibly adopted to the similar water-level monitoring and controlling systems.

## 3. Material and Methods

### 3.1. Method Overview

The deep autoencoder is an excellent artificial neural network used to learn efficient data encoding in an unsupervised fashion. The main technique of autoencoder intends to learn a latent representation for a collection of data $X=\{x_{t=1},x_{t=2},x_{t=3},\cdots ,x_{t=T}\}$, assuming $x_{t=1}\in R^{S}$, using encoding and decoding, where *S* is segment length. That way, the neural network learns the most distinctive features from the data. After the decoding, we can compare the input to the output and examine the difference by computing MSE (see Equation (2)). If there is a considerable distinction (the reconstruction loss is higher than a defined threshold), we can assume that the model struggled to reconstruct the data, and thus, this data point falls under the doubt of being an anomaly.

**Figure 1:**
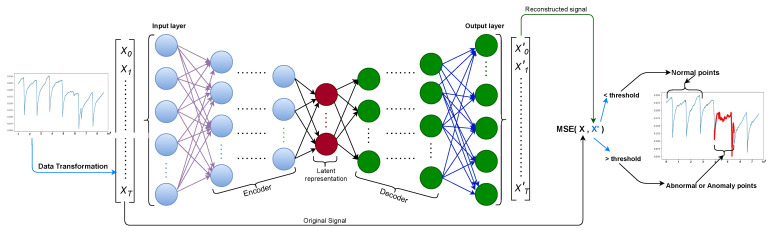
Anomaly detection pipeline using deep autoencoder-based neural network model.

Figure 1 shows the steps followed to capture anomaly points in the given data. In the figure, we can see that the deep autoencoder model is composed of input, encoder, latent space representation (bottleneck), decoder, and output layers. Consequentially, deep autoencoder can be constructed by extending the encoder and decoder component with multiple hidden layers. The input layer accepts the input that passed through data normalization to rescale the data to a specific interval, followed by sliding window to produce the sub-sequence of the continuous input, then down-sampling to preserve significant information from the big size sub-sequence of the input data. Section 3.3.2 and Section 3.3.3 cover detailed explanations of each component of the data preprocessing pipeline.

The encoder, this part of the network compresses the input in a reduced dimension into a latent space representation. The compressed sequence is the compacted version of the original sequence.

The latent space representation or the bottleneck layer is part of the network which represents the compressed input that goes to the decoder. The well-designed bottleneck layer learns to distinguish and decide the relevant features of the data to keep and discard other aspects. This learning process is governed by the compactness of representation, measured as the compressibility, and preserves some behaviorally relevant variables from the input.

The decoder layer translates the encoded sub-sequence back to the original dimension. The decoded output is a lossy reconstruction of the original input, which is reconstructed from the latent space representation.

### 3.2. Data Description

In this section, we will briefly explain the data collection process and introduce the datasets we use to perform our experiments. Experiments in this work used several time-series datasets from the sensor sites named ‘PipelineCorridor’ and ‘UtilityCorridor’. The site name PipelineCorridor came from pipeline corridor because it refers to pipeline pathways or corridor within which the pipeline which transmit liquid or gas are located. And UtilityCorridor came from Utility corridor because it refers to linear alignment location of a utility such as stormwater, wastewater, water, communication lines or electric. These two datasets were collected and provided by an IT-based infrastructure company, Infranics, located in Seoul, South Korea. Infranics gathers data from several sensor devices and provides solutions such as intelligent safety analysis and prediction services for safety supervision on underground communication systems.

**Figure 2:**
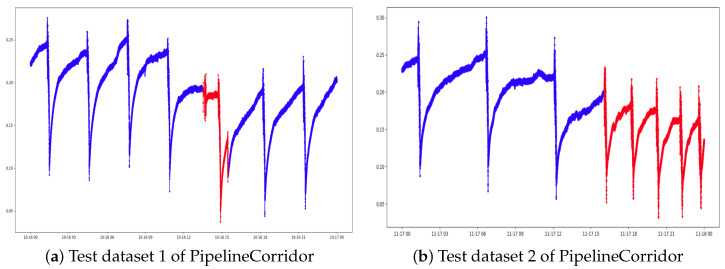
Test datasets used for evaluating the performance of the anomaly detection models.

To collect the data, the sensors were wired on the infrastructure’s surroundings to attain a real-time reading of the water level. The readings were grouped in several datasets to be used in this work. Those datasets are one to four weeks of operative data captured at an interval of one second. Table 1 summarizes the data information and data splits for training and performance evaluation of the models for time-series anomaly detection system (explained in Section 3.1). And Figure 2 shows the portions of the PipelineCorridor datasets we used to evaluate the anomaly detection model.

### 3.3. Preprocessing Pipeline

**Figure 3:**
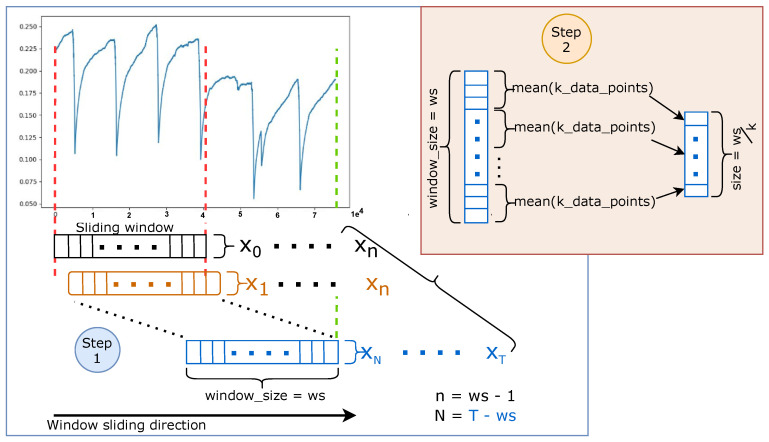
Data preprocessing pipeline, step 1 reproduces the data by stepping over the data in a specific direction and a fixed step size, then, step 2 receives a data from step one and down-samples the data to the model input size.

To efficiently train neural network-based anomaly detectors with time-series data, data preprocessing for experiments is a crucial process. The preprocessing pipeline combines a series of steps to transform the time-series data input and compresses them into a representation suitable for applying deep learning models. Figure 3 shows the two steps for data replication in the data preprocessing pipeline: sliding window and down-sampling. The specific configuration of this pipeline includes the exploratory data analysis (EDA) [37] and preprocessing steps. EDA provides a way of understanding different data aspects through visualization techniques (histograms, box plots, scatter plots, graphs etc.) and correlation analysis, bringing out the data parameters’ relationship and other relevant information. Through EDA, analysts can gain knowledge of the data properties and attempt to grasp useful information extracted from the data, which will guide appropriate selection of data preprocessing tools and techniques.

In this work, we started by replacing the timestamps with the increasing integers of 1 for both datasets (training and test datasets), where each data point represents 1 s worth of data. Then we preprocessed the data by following the data pipeline preprocessing procedures described in Section 3.3.1, Section 3.3.2, Section 3.3.3 and Section 3.3.4.

#### 3.3.1. Data Normalization

Normally, data normalization adds some benefits for the convergence of the model as discussed by LeCun et al. [38]. There is a varieties data normalization techniques [39] that can be applied during data preprocess. We used a minmax normalizer to rescale features to intervals 0 to 1 linearly in our preprocessing pipeline. Specifically, to rescale values to a required interval, we computed the difference between each data value and the minimum value in the given dataset divided by the difference of maximum and minimum values. Equation (1), which is for data transformation, summarizes the minmax normalizer.

(1) $$x_{new}=\frac{x_{i}-x_{min}}{x_{max}-x_{min}}$$

The $x_{min}$ and $x_{max}$ values obtained from the training data are the same values applied during test data normalization.

#### 3.3.2. Sliding Window Approach

A sliding window [40] approach is used to transform a continuous time-series into sub-sequences or discrete sequences as depicted in step 1 in Figure 3. The window size ws corresponds to the length of the resulting sequence, and the stride size can be adjusted. For example, a sequence $t_{0},t_{1},t_{2},t_{3},t_{4},t_{5},t_{6},t_{7}$ with sliding window size of 6 and stride of 1 outputs a set of sequences $\left\{\left\{t_{0},t_{1},t_{2},t_{3},t_{4},t_{5}\right\},\left\{t_{1},t_{2},t_{3},t_{4},t_{5},t_{6}\right\},\left\{t_{2},t_{3},t_{4},t_{5},t_{6},t_{7}\right\}\right\}$.

#### 3.3.3. Data Compression

Down-sampling, as depicted in step 2 in Figure 3, further compresses the time-series data to a sequence of data points that matches our model input requirements. For the compression of the sequence with the length ws (window size), we use a mean function as an aggregation function. First, we set an integer value *k* as a statistical measurement to further divide the sequence of size *ws* into *S* segments. Then the mean function computes the average of each segment thus results in a vector of $S=\frac{ws}{k}$ features. Please note that if a statistical measurement is set too large, down-sampling will lead to loss of the relevant information. On the other hand, if the statistical measurement is set too low, then the compressed data may be still inefficient for processing because the difference from the compression is marginal.

#### 3.3.4. Sequence Labeling

The task of labeling each sequence with metadata is basically encoding additional relevant information concerning the input data. Please note that we have used normal data for training our models; however, each observation in the test data already contains the label (1 for normal and −1 for abnormal). Moreover, the test data also need to undergo the preprocessing in Figure 3, and then, we need to relabel the resulting test sequences as either normal or abnormal. Therefore, we assign a value of −1 as the label of the test sequence if any observations inside the test sequence contain the value other than 1; otherwise, we simply assigned a value of 1. In our experiment, we perform this relabeling after the data preprocessing phase.

### 3.4. Detection Accuracy

We have explained how our approach performs the reconstruction (in Section 3.1) and the data pipeline preprocessing (in Section 3.3), and we need to explain how anomalies are detected based on the data reconstruction error. More specifically, when the autoencoder model generates a reconstruction of the input data, we compute the error, which is the difference between the input data and its reconstructed version. In our work, the error is the value computed by the mean squared error (MSE) (see Equation (2)) for each input sequence.

(2) $$e_{X_{t},{\hat{X}}_{t}}=\frac{1}{S}\sum_{i=0}^{S-1}{\left(x_{i}-{\hat{x}}_{i}\right)}^{2}$$

In Equation (2), $e_{X_{t},{\hat{X}}_{t}}$ is the MSE value of the sequence $X_{t}$ against ${\hat{X}}_{t}$, the $X_{t}$ is the input vector of dimension $R^{S}$ (thus, $x_{i}\in X_{t}$), and ${\hat{X}}_{t}$ is the sequence generated by the trained model given the data input vector $X_{t}$ (thus, ${\hat{x}}_{i}\in {\hat{X}}_{t}$).

Consequently, we are relying on the reconstruction error and the threshold that can discriminate between anomalous and normal data points to capture substantial amount of anomaly data points, and we computed MSE using Equation (2). For the calculation of the threshold, we adopted three-sigma rule expressed in Equation (3).

(3) $$threshold=mean\left(loss_{X_{train}}\right)+3\times std\left(loss_{X_{train}}\right)$$

In Equation (3), the mean function computes the mean of the training prediction loss (i.e., $loss_{X_{train}}$) distribution and *std* is the standard deviation function which returns a standard deviation of the training loss distribution.

We used Equation (4) to label each data point from model prediction. This equation compares the reconstruction error of the sequence, $e_{X_{t}}$ against the computed threshold (see Equation (3)) and returns label for the given sequence of the data points $X_{t}$.

(4) $$l_{X_{t}}=\theta \left(e_{X_{t}}\right)=\left\{\begin{array}{cc} +1 & \mathrm{if}\;e_{X_{t}}\;<\;threshold\\ -1 & \mathrm{otherwise} \end{array}\right.$$

where $l_{X_{t}}$ is a predicted label assigned to the sequence $X_{t}$ which is either of value 1 for normal or −1 for abnormal data points. A function θ returns a value as a label after applying the selected threshold to the reconstruction error.

### 3.5. Evaluation Metric

For the accurate and effective performance evaluation of our anomaly detection system, we used a confusion matrix from our experimental results to compute the fundamental classification metrics including accuracy, recall, precision, and F1-score. The confusion matrix computed is a 2D matrix as shown in Table 2. With two labeled sets (actual or true and predictions), we can create a confusion matrix that will summarize the results of evaluation or inference phase of the classifier.

From the computed confusion matrix, we further computed the accuracy (Equation (5)), precision (Equation (6)) recall (Equation (7)), and F1-Score (Equation (8)) as follows:

(5) $$Accuracy=\frac{TP+TN}{TP+FP+FN+TN}$$

(6) $$Precision=\frac{TP}{TP+FP}$$

(7) $$Recall=\frac{TP}{TP+FN}$$

(8) $$F1-score=2\times \frac{Precision\times Recall}{Precision+Recall}$$

Please note that the F1-score (in Equation (8)) is a number between 0 and 1 and is the harmonic mean of precision and recall.

#### Receiver Operating Characteristics

We employed ROC as another metric to further evaluate different model performances on different datasets. A ROC curve is a plot of the false positive rate (1-Specificity) in the function of the true positive rate (Sensitivity) for different threshold values (cut-off points) of a parameter. Each point on the ROC curve represents a sensitivity and specificity pair corresponding to a specific decision threshold. The classifier’s performance on binary problems can be measured using Area Under the Curve (AUC) in the ROC curve.

### 3.6. Experimental Setup

In this section, we briefly explain the implementation detail for our neural network models. We set up our experiment as follows; we implemented our deep autoencoder models using the Sequential model of Keras API. We used the MSE loss function (in Equation (2)) with Adam optimizer [41], and we set the learning rate to a fixed value of 0.001. Each of deep autoencoder models consists of an input and an output layer with *n* hidden layers with rectified linear units. We trained each model with mini-batches for 50 epochs. The size of mini-batches ranged from 10 to 50 instances, which is chosen based on the model complexity.

### 3.7. Training and Evaluation Procedures

To experiment on the data and investigate the model architectures for the specific configurations, we applied two different training-evaluation procedures, which are epoch-wise training-validation and walk-forward validation. By epoch-wise training-validation, we are referring to the standard training and testing procedure in which a model is trained on a portion of the data and is tested on an unseen portion. First, we fit the model to the training data for several epochs until our stopping criterion is satisfied. After that, we perform the model evaluation to check how accurately the predictive model can perform on the unseen data, which is necessary to ensure the model is ready for deployment.

On the contrary, in the walk-forward validation procedure, we use window-sliding technique to repeatedly split the dataset into the train and validation sets. Then from each set, we fit a model on the train data (that is $x_{i\times d}$ to $x_{i\times d+ws-1}$ data points), then we evaluated its performance on validation data (that is $x_{i\times d+ws}$ to $x_{i\times d+ws+m}$ data points). The ws is training data window size, *d* is the stride, *m* is the validation data window size, and *i* is the index of validation steps. The values of ws, *d* and *m* are hyperparameters set to integer values. By incrementing *i* (initialized as zero), we move the window with a stride size *d* after each iteration of training and validation loop, until (i×d+ws+1)>D, where *D* is the number of time-series data points in the given dataset. For the optimal results, we chose ws≥2d to allow the model to be exposed to sufficient data points and to acquire data dynamics with different and desired patterns.

## 4. Results and Discussion

For the evaluation and the analysis of our methods, we conducted systematic experiments using two procedures discussed in Section 3.7 and evaluation metrics discussed in Section 3.5. For the detailed discussion of the experiments and their results, we divided this section into two parts, which are epoch-wise training-validation analysis and walk-forward validation analysis.

### 4.1. Part 1: Epoch-Wise Training-Validation Analysis

**Figure 4:**
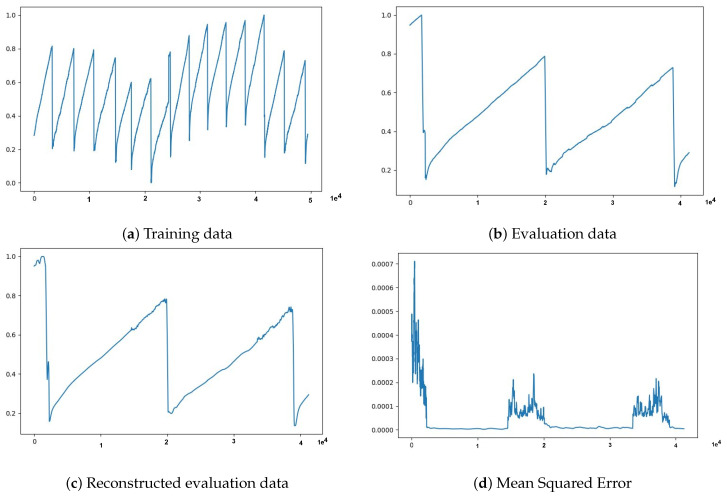
The data used were collected for 24 h for 7 days, normalized to be in the range of 0 to 1 and then divided into training and evaluation datasets. Training data in (**a**) depicts six days of data points, evaluation data in (**b**) depicts one day (i.e., 7th day) data points, (**c**) shows model output represents generated sequence data, and, in (**d**), the MSE computed between input and model generated output is plotted.

We applied epoch-wise training-validation procedure to prepare the model and evaluate its reconstruction capability on normal data only, after that we worked with data containing both normal and abnormal data points. We first tried to use Sensor data 1 to Sensor data 4 datasets from the UtilityCorridor site by splitting each dataset into two parts, six(6) days of the data for training and 7th day of the data for evaluation. In these experiments, we trained the models on the train data and tracked the reconstruction error on the evaluation data to see how well the model can reconstruct the unseen data. The experiment results, shown in Figure 4, indicate that the model can regenerate out-of-sample data with a minimum and reasonable mean squared error.

**Figure 5:**
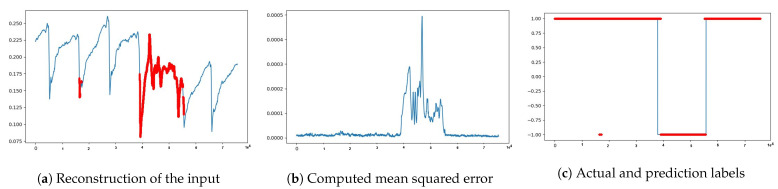
Visualization of model reconstruction output along with the red colored anomaly predictions in (**a**), MSE’s between the inputs and the predictions to capture anomalies are in (**b**) and true labels in blue color followed by the predictions in red color are in (**c**).

Now that the experimental results in Figure 4 demonstrated the potential capability of our approach, we performed extensive experiments with further anomaly detection tasks. We started with three(3) h of data equivalent to a window size ws of 10,800 data points and a statistical measurement *k* of 60 s. That means 10,800 data points result in 180 features after down-sampling (i.e., 180 = 10,800/60), which is the input and output size of our deep autoencoder. The results of the experiment are summarized in Figure 5 and in Table 3, where the entries are computed based on the evaluation metrics described in Section 3.5.

From the experiment results in Table 3, we can see that neurons play an essential role in the performance improvement of the neural network model. As shown in Table 3 $model_{2}$ outperformed $model_{1}$ while it has only four(4) more neurons than that of $model_{1}$ for bottleneck layer (latent representation layer). The number of neurons was the only hyperparameter changed to lead to this big performance margin in all tasks. However, both the models seem to be too biased on the training data to cope with slight domain shift. In other words, when the model trained on the Train data 1 performs well on the Test data 1 but poorly on the Test data 2 and the same goes when trained on the Train data 2 and evaluated on the Test data 1 and the Test data 2. This can obviously seen from $model_{2}$ performance in Table 3. However, when we trained models on combined datasets (i.e., the Train data 1 and Train data 2), the models somehow showed similar performance on both out-of-sample data.

The experiment results elicited several considerations that required further investigation, which were related to model performance on different data patterns and sizes. These considerations were the amount of data for training, input size, various statistical measurements, training and testing procedures such as walk-forward validation (see Section 3.7) that will be discussed in Section 4.2.

### 4.2. Part 2: Walk-Forward Validation Analysis

**Figure 6:**
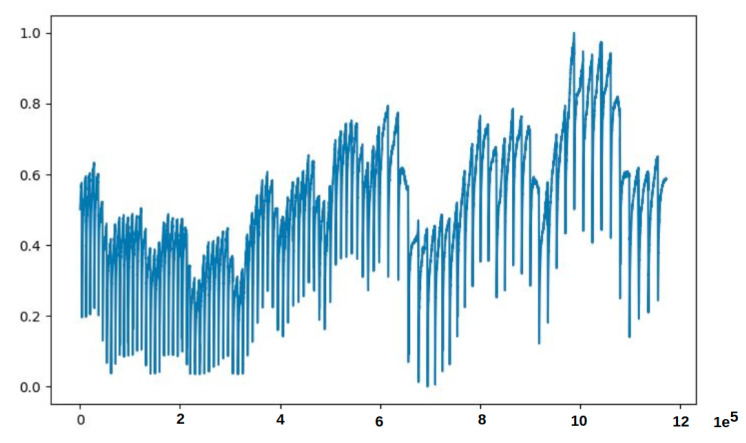
Two weeks preprocessed data of PipelineCorridor ready to train the model. The data contains some points which are out of range from most of the data. Please note that each value on the *x*-axis corresponds to a timestamp in 1 s, and each value on the *y*-axis is the normalized data point.

We employed the walk-forward validation procedure to improve the model performances over time while preserving the temporal nature of the data. More precisely, we investigated the model capabilities over time with several considerations such as different window sizes (including 10,800, 18,000, 25,200, and 36,000), statistical measurements of 60 and 120 s (see Table 4) and the amount of data ranged from 7 days of data to 14 days of data (see Figure 6).

We preprocessed the data following the data preprocessing pipeline (in Section 3.3) and then we ran several experiments for different configurations as we described in the beginning of the Part 2. The number of model versions we can obtain from each experiment is equivalent to the number of iterations (i.e., total sliding window moves). Then we performed evaluation of each model using the transformed Test data 1 and Test data 2 datasets. Then we computed the accuracy, precision, recall, and F1-score. Please note that we bold some of the values in the experiment result tables (Table 5, Table 6 and Table 7) to indicate the highest performance results achieved by the models.

After performing the model evaluations and obtaining predictions, we analyzed all the output and drew two useful observations to improve model performance.

**Figure 7:**
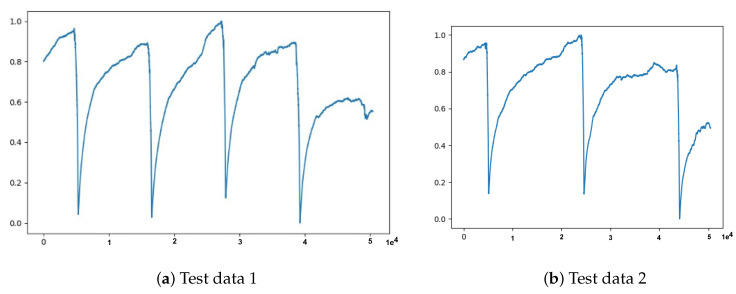
Illustrations of test data transformed using window size of 36,000 and statistical measurement of 120 s through preprocessing step.

**Figure 8:**
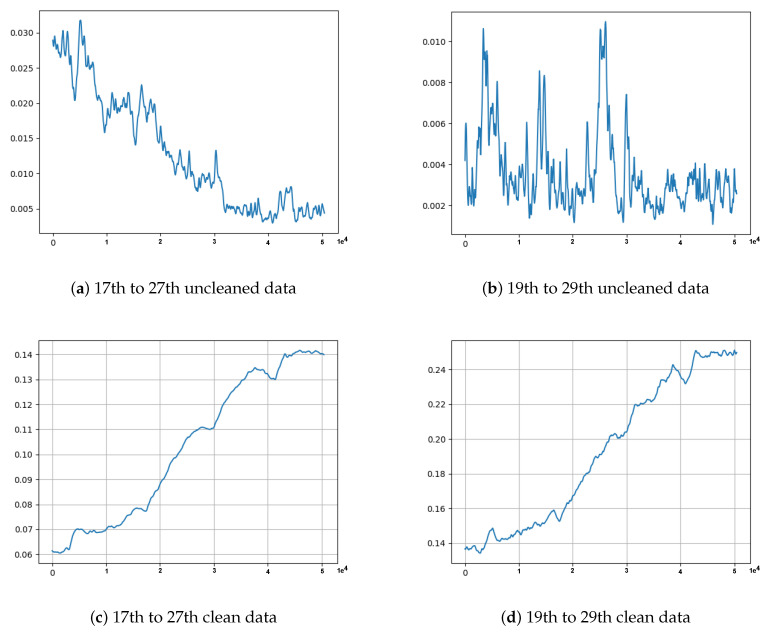
Please note that (**a**–**d**) are MSE graphs computed after evaluating trained models on Test data 1. A value on the *x*-axis is a timestamp down-sampled by 2 min. In (**a**,**c**), the models were trained on 17th to 27th data, and in (**b**,**d**), the models were trained on 19th to 29th data. The training data for models in (**a**,**b**) were uncleaned data (i.e., data contains minority data points that are out of range), while the training data for (**c**,**d**) were clean data (i.e., data without minority (out of range) data points). The statistical measurement of 120 s was used for down-sampling all data in these experiments.

**Figure 9:**
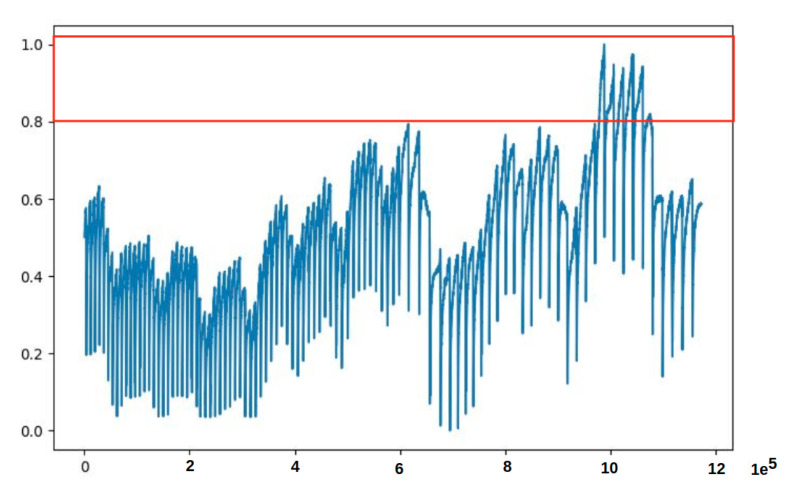
Uncleaned data containing minority data points within the red box detected during model output analysis. A value on *x*-axis is a timestamp of 1 s, and each value on the *y*-axis is the normalized data point.

**Figure 10:**
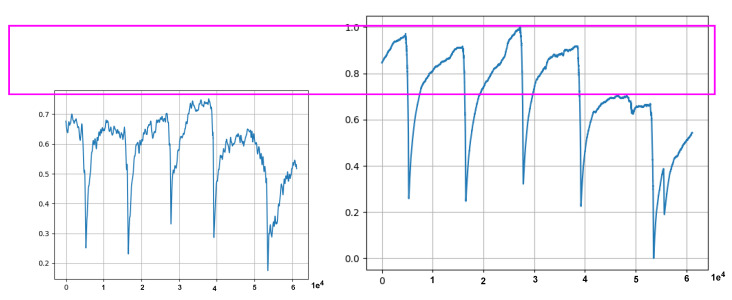
Model generated data (on the **left**) that deviates from the expected distribution due to the effect introduced by the training data. The pink rectangle shows the discrepancies between the generated data and the original Test data 1 (on the **right**). A value on the *x*-axis represents the timestamp down-sampled by 1 min, and a value on the *y*-axis is the mean value of 60 data points.

1. The first observation was the problem innate in the training data. Some minority data points found in the training data had caused the model to fail to reconstruct the input (Test data 1 in Figure 7a and Test data 2 in Figure 7b), and hence failed to correctly identify anomaly data points. This phenomenon is depicted in the MSE graphs in Figure 8. Please note that in Figure 8, we can see that the MSE’s in (a) and (b) do not correspond to (actually, are completely opposite to) the MSE’s in (c) and (d). From Figure 9, we can see that the unclean training data contains some minority data points (within the red box). The MSE’s calculated from the 17th to 27th part of the unclean training data (in Figure 9) are drawn in Figure 8a, and those from the 19th to 29th part are drawn in Figure 8b.
2. The second observation was the discrepancy (indicated by a violet-colored box in Figure 10) between the original Test data 1 in Figure 8a and its corresponding reconstructed data. It was because the majority values in the training data were within the range of 0.0 to 0.8, which forced the model to try to reconstruct other values, which are larger than 0.8, to be fit within the range of the majority values.

**Figure 11:**
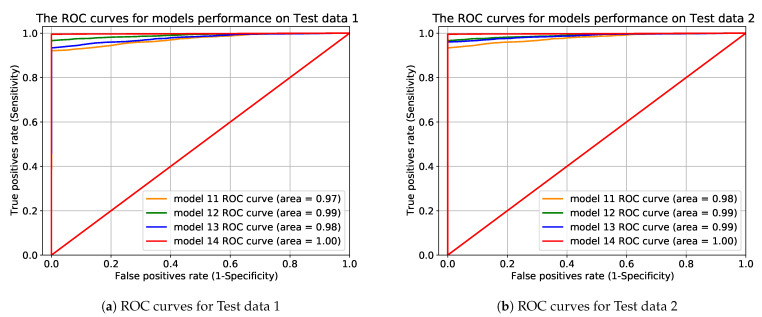
The ROC curves for performance comparison for *model*_11_, *model*_12_, *model*_13_, and *model*_14_ when models were trained on 10 h of data and then tested on Test data 1 in (**a**) and Test data 2 in (**b**).

To mitigate the problematic data effects, we removed the portion of minority (out of range) data from training data. After removing those minority data points and exploring the considerations including the amount of training data, input size, different statistical measurements and training procedures, we found that the performance of the model were drastically improved as shown in Table 5, Table 6 and Table 7 and in the ROC curves in Figure 11.

## 5. Conclusions

In this paper, we have proposed deep autoencoder technique for anomaly detection. Our implementation for water-level anomaly detection combined state-of-the-art deep learning and data preparation routines. We adopted those techniques based on their compatibility, simplicity, and capability to learn complex functions to represent an optimal subspace for normal data.

We investigated different window sizes, including 10,800, 18,000, 25,200, 36,000, and statistical measurements of 60 and 120 s. In addition, we tuned combinations of hyperparameters to find the best fit for each experiment configuration. Our autoencoder technique achieves the excellent result of 99.9% F1-score and 1.00 AUC when we used a window size of 36,000 and statistical measurement of 120 s. Our proposed methodology has proven its effectiveness in all settings despite the data complexity with a simple threshold selection procedure for anomaly detection.

## Figures and Tables

**Table 1:** Sensor site in the first column, dataset name in the second column, the number of data points in each dataset in the third column and the indicator of presence of labels in the last column “YES” indicates that the file contains labels and “NO” indicates that the file does not contain labels.

Sensor Site	Dataset Name	# of Data Points	Presence of Labels
PipelineCorridor	Train data 1	604,767	NO
	Train data 2	604,775	NO
	Test data 1	86,400	YES
	Test data 2	86,396	YES
PipelineCorridor	4 weeks	2,419,100	NO
UtilityCorridor	Sensor data 1	81,972	NO
	Sensor data 2	86,396	NO
	Sensor data 3	86,396	NO
	Sensor data 4	86,396	NO

**Table 2:** The representation of the confusion matrix for binary classification problem. True Positive (TP): both actual and prediction are positive (agree), False Positive (FP): actual class is negative, but prediction is positive (disagree), False Negative (FN): actual class is positive, but prediction is negative (disagree) and True Negative (TN): both actual class and prediction are negative (agree).

	Predicted–Positive	Predicted–Negative
Actual–Positive	TP	FN
Actual–Negative	FP	TN

**Table 3:** Precision, recall and F1-score for model performances evaluation during data and model architecture investigations. Please note that $model_{1}$ has slightly fewer number of neurons than $model_{2}$ in the latent representation layer.

	${\mathrm{model}}_{1}=180\times 70\times 50\times 5\times 50\times 70\times 180$			${\mathrm{model}}_{2}=180\times 70\times 50\times 9\times 50\times 70\times 180$		
	**Recall**	**Precision**	**F1-Score**	**Recall**	**Precision**	**F1-Score**
**Train data 1**						
Test data 1	0.918	0.982	0.622	0.907	0.988	**0.946**
Test data 2	0.365	0.892	0.281	0.422	0.401	**0.411**
**Train data 2**						
Test data 1	0.547	0.603	0.348	0.626	0.700	**0.6612**
Test data 2	0.650	0.856	0.556	0.915	0.878	**0.896**
**Combined**						
Test data 1	0.603	0.978	0.464	0.676	0.982	**0.801**
Test data 2	0.699	0.683	0.401	0.741	0.934	**0.826**

**Table 4:** Down-sampling hyperparameters and the input/output sizes for both training and test datasets, the first column shows the window size in hours per each experiment.

	Down-Sampling Variables Size		
	**Window Size**	**Statistical Measurement**	**Input/Output Size**
5 h of data			
	18,000	60 s	300
	18,000	120 s	150
7 h of data			
	25,200	60 s	420
	25,200	120 s	210
10 h of data			
	36,000	60 s	600
	36,000	120 s	300

**Table 5:** Precision, recall and F1-score for model performances evaluation during data and model architecture investigations after removing minority (out of range) data. All models were trained on five hours of data with two different statistical measurements.

	${\mathrm{model}}_{3}=150\times 90\times 60\times 90\times 150$			${\mathrm{model}}_{4}=150\times 100\times 60\times 100\times 150$		
	**Recall**	**Precision**	**F1-Score**	**Recall**	**Precision**	**F1-Score**
**statistical measurement—120 s**						
Test data 1	0.905	0.100	0.950	0.937	1.000	**0.967**
Test data 2	0.988	0.952	0.970	0.922	0.957	**0.940**
	${\mathrm{model}}_{5}=300\times 200\times 60\times 200\times 300$			${\mathrm{model}}_{6}=300\times 200\times 100\times 200\times 300$		
	Recall	Precision	F1-score	Recall	Precision	F1-score
**statistical measurement—60 s**						
Test data 1	0.958	0.874	0.914	0.987	0.984	**0.991**
Test data 2	0.960	0.989	**0.974**	1.000	0.949	**0.974**

**Table 6:** Precision, recall and F1-score for model performances evaluation during data and model architecture investigations after removing minority (out of range) data. All models were trained on 7 h of data with two different statistical measurements.

	${\mathrm{model}}_{7}=210\times 150\times 60\times 150\times 210$			${\mathrm{model}}_{8}=210\times 160\times 90\times 160\times 210$		
	**Recall**	**Precision**	**F1-Score**	**Recall**	**Precision**	**F1-Score**
**statistical measurement—120 s**						
Test data 1	0.915	0.950	0.932	0.985	0.965	**0.975**
Test data 2	0.937	1.000	0.968	1.000	0.996	**0.998**
	${\mathrm{model}}_{9}=420\times 210\times 60\times 210\times 420$			${\mathrm{model}}_{10}=420\times 210\times 90\times 210\times 420$		
	Recall	Precision	F1-score	Recall	Precision	F1-score
**statistical measurement—60 s**						
Test data 1	0.937	0.827	0.879	0.987	0.984	**0.991**
Test data 2	0.839	0.986	0.913	0.993	0.991	**0.992**

**Table 7:** Precision, recall and F1-score for model performances evaluation during data and model architecture investigations after removing minority (out of range) data. All models were trained on 10 h of data with two different statistical measurements.

	${\mathrm{model}}_{11}=300\times 150\times 60\times 150\times 300$			${\mathrm{model}}_{12}=300\times 190\times 90\times 190\times 300$		
	**Recall**	**Precision**	**F1-Score**	**Recall**	**Precision**	**F1-Score**
**Statistical Measurement—120 s**						
Test data 1	0.987	0.956	0.971	0.994	1.000	**0.997**
Test data 2	0.985	0.965	0.975	0.998	1.000	**0.999**
	${\mathrm{model}}_{13}=600\times 190\times 90\times 190\times 600$			${\mathrm{model}}_{14}=600\times 200\times 100\times 200\times 600$		
	Recall	Precision	F1-score	Recall	Precision	F1-score
**Statistical Measurement—60 s**						
Test data 1	1.000	0.979	0.989	1.000	0.992	**0.996**
Test data 2	1.000	0.986	0.993	0.993	1.000	**0.996**

## Data Availability

The data that support the findings of this study are available from Infranics Co., Ltd., but restrictions apply to the availability of these data, which were used under license for the current study, and so are not publicly available. Data are however available from the authors upon reasonable request and with permission of Infranics Co., Ltd.

## Abbreviations

The following abbreviations are used in this manuscript:

AUC	Area Under the Curve
EDA	Exploratory Data Analysis
LSTM	Long Short-Term Memory
MDPI	Multidisciplinary Digital Publishing Institute
MSE	Mean Squared Error
PCA	Principal Component Analysis
ROC	Receiver Operating Characteristics
SVM	Support Vector Machine
